# Characteristics and outcome of microsporidial keratitis

**DOI:** 10.1186/s12348-025-00506-5

**Published:** 2026-04-13

**Authors:** Khalid Alburayk, Abdullah Alfawaz, Khalid Tabbara

**Affiliations:** 1 Research Excellence Center in Ophthalmology and Visual Sciences, Department of Ophthalmology, College of Medicine, King Saud University, Riyadh, Saudi Arabia; 2 King Saud University Medical City, Riyadh, Saudi Arabia; 3 Eye center, Riyadh, Saudi Arabia

**Keywords:** Cornea, Microsporidial, Keratitis, Infection, Honey

## Abstract

**Purpose:**

Microsporidia are a diverse group of obligate, intracellular, eukaryotic, spore-forming fungi like and not typical true fungi. Ocular manifestations vary from early to late stages of infection and include keratoconjunctivitis, stromal keratitis, scleritis, and rarely endophthalmitis. It is rarely reported in ophthalmology literature. The purpose of this study is to evaluate the characteristics and outcome of 26 cases of microsporidial keratitis.

**Methods:**

We retrospectively reviewed the medical records of 26 immunocompetent patients with microsporidial keratitis presented in the period between 2007 and 2024. The inclusion criteria were all patients with confirmed diagnosis of microsporidial keratitis based on the clinical symptoms and confirmation of diagnosis that was done by microscopic identification of microsporidial organisms in corneal or conjunctival scrapings.

**Results:**

The study included 26 patients (mean age: 39 years), with a male predominance (65%). Most were initially misdiagnosed, with epithelial or bacterial keratitis being the most common preliminary diagnoses. The most frequent presentation was diffuse epithelial keratitis (58%), followed by stromal infiltrates and keratoconjunctivitis. Notably, 53% of patients had a history of topical steroid use, and 23% reported prior application of unsterile topical honey. Visual acuity at presentation varied, with 27% presenting at 20/200 or worse. Visual outcomes improved over time, with patients presenting with diffuse epithelial keratitis showing the most favorable recovery. Stromal and disciform keratitis presentations were associated with poorer visual outcomes at 1 month.

**Conclusion:**

Microsporidial keratitis is frequently misdiagnosed, leading to delayed treatment. Diffuse epithelial keratitis was the most common presentation and associated with better visual outcomes, while stromal involvement correlated with poorer prognosis. Early diagnosis and appropriate management are essential to optimize visual recovery.

## Introduction

Microsporidia are a diverse group of obligate, intracellular, eukaryotic, spore-forming recently reclassified as fungi [1, 2]. Ocular manifestations include keratoconjunctivitis, stromal keratitis, scleritis, and in rare cases, endophthalmitis [3, 4]. Since their discovery in silkworms in 1857, there have been approximately 200 genera and over 1700 species of microsporidia reported [5, 6]. The exact mechanism of microsporidial corneal infections remains unknown. However, as microsporidia are waterborne pathogens, exposure to contaminated water has been reported as a potential risk factor [7, 8]. Sabhapandit et al., reported that 47% of patients had a history of eye injuries [9]. Additionally, wearing contact lenses poses a significant risk, as they may facilitate the entry of the organism into the cornea by serving as a conduit. In rare cases, corneal infections have developed following eye surgeries [10]. Microsporidia can also infect bees, which can lead to the death of the bee and subsequent spread of the infection to the honey.

The most effective medical course of treatment for microsporidial keratitis remains unclear. Various medications including, albendazole, thiabendazole, itraconazole, fumagillin, propamidine isethionate, chlorhexidine, metronidazole, polyhexamethylene biguanide, benzimidazoles, and fluconazole, were previously used to treat microsporidial keratitis. This study aimed to evaluate the course of the disease and outcome in 26 patients diagnosed with microsporidial keratitis. Although microsporidiosis is increasingly recognized as an opportunistic infection, especially in immunocompromised individuals, there remains a lack of clarity in several key areas. First, the clinical presentation of microsporidial infections is not well-defined, with a wide range of nonspecific symptoms that can overlap with other infections. Second, treatment protocols vary across settings, and there is no universally accepted standard of care. Third, the current classification systems for microsporidia are often inconsistent or outdated, making diagnosis and reporting challenging. Finally, there is limited data on the long-term clinical outcomes of patients diagnosed with microsporidiosis, particularly in relation to different management strategies.

Our study aims to fill these gaps by systematically collecting and analyzing the clinical presentations of confirmed microsporidia cases and evaluating the treatment approaches and outcomes.

## **Materials and method**s

We retrospectively reviewed the medical records of 26 immunocompetent patients with microsporidial keratitis between 2007 and 2024. Inclusion criteria were all patients with a confirmed diagnosis of microsporidial keratitis, characterized by clinical symptoms of redness, pain, tearing, blurry vision, and clinical findings such as disciform keratitis, epithelial keratitis, corneal ulcer, or diffuse epithelial keratitis. The diagnosis was confirmed by identifying microsporidial organisms in corneal or conjunctival scrapings using light microscopy with Giemsa stain, which showed intracellular spores, cytoplasmic vacuolation, and increased surrounding tissue cellularity. The spores typically stain blue with Giemsa, contrasting with the pink-staining cytoplasm of the host cell. All patients were treated with topical fumagillin 3 mg/mL with topical fluconazole 2% every 2 h for 1 week, followed by every 6 h for 1 month. The primary outcome measured was visual acuity outcome. Secondary outcomes included symptom relief, resolution of keratitis, subsequent scarring, and recurrence of infection.

Data were compiled, verified, and analyzed using the Statistical Package for Social Sciences (SPSS Inc., Chicago, IL, USA) version 26. For each attribute and demographic variables, descriptive statistics (i.e., frequencies, percentages, and measures of central tendency and dispersion, where applicable) were generated. A comparison was made using the Chi-square test, and a paired test was conducted to assess the significance of visual acuity improvement pre- and post-treatment at 1, 3, and 6-month follow ups. A *p*-value < 0.05 was considered statistically significant.

This study was approved by the Institutional Review Board of King Saud University, College of Medicine, Riyadh, Saudi Arabia. The authors certify that all applicable institutional and governmental regulations regarding the ethical use of human volunteers/animals were adhered to during this research.

## Results

### Patients characteristics

Table 1 presents a comprehensive summary of the study participants’ characteristics and medical history. The mean age was 39 years, ranging from 19 to 60 years. The sample comprised 35% females (*n* = 9) and 65% males (*n* = 17). All patients were referred to our center with different clinical diagnoses from several ophthalmologists, Most patients (80%) were initially misdiagnosed, with the most common initial diagnosis being unspecified epithelial keratitis followed by bacterial keratitis and Herpetic eye disease. Over half of patients were diagnosed correctly after a 1 month delay (55%; Table 1).

**Table 1:** Summary of patients characteristics

Categories	*N*	% out of 26 eyes
Mean age ± SD (Range)	26	39.85 ± 13.25 (19–60)
Female	9	35%
Male	17	65%
Unilateral	16	62%
Bilateral	10	38%
Initial referral diagnosis	21	80%
Acanthamoeba keratitis	1	4.8%
Fungal keratitis	1	4.8%
Dry eye	1	4.8%
Epithelial keratitis	5	19.0%
HSV	2	9.5%
HZV	1	4.8%
Medicamentosa	2	9.5%
Bacterial Keratitis	3	14.3%
Delay in diagnosis	18	69%
<= 1 month	8	44%
> 1 months	10	56%
Usage of Honey as remedies	6	23%
Usage of Topical steroid	14	54%

### Presentation and significant history

The most common initial presentation was diffuse epithelial keratitis (58%), followed by stromal infiltrate and keratoconjunctivitis. The least common presentations were persistent epithelial defects and a disciform keratitis like picture. Furthermore, almost half of patients (53%) used topical steroids in variable preparations for different reasons. Moreover, about one quarter of patients (23%) reported prior use of unsterile topical honey as a herbal remedy before developing the keratitis (Table 2).

**Table 2:** Summary of patients characteristics

Presentation	26	100%	Old classification	Proposed new classification
Diffuse epithelial keratitis	15	57.7%	Keratoconjunctivitis	Epithelial
Disciform keratitis	2	7.7%	Stromal keratitis	Endothelial
Keratoconjunctivitis	3	11.5%	Keratoconjunctivitis	Epithelial
Persistent epithelial defect	2	7.7%	Keratoconjunctivitis	Epithelial
Stromal infiltrate	4	15%	Stromal keratitis	Stromal

### Visual acuity

Initial visual acuity varied significantly among patients, as 27% of participants presented with 20/200 vision or worse and 35% of patients presented with vision ranging from 20/40 to 20/125. Finally, 38% of patients presented with vision ranging from 20/20 to 20/40. Visual acuity improved over the 1-, 3-, and 6-month follow-up periods (Table 3).

**Table 3:** Summary and comparison of visual acuity at 1-, 3-, and 6-month follow ups with visual acuity at presentation

Time (number of patients)	Mean(dec)	SD(dec)	Mean (Snellen)	Paired t-test (*p*-value)
Visual Acuity on presentations	0.48	0.36	20/40	N/A
One Month	0.72	0.25	20/28	0.008
Three Month	0.69	0.33	20/29	0.006
Six Month	0.76	0.34	20/26	0.007

Table 4 presents the visual outcomes of each presentation, which showed a significant association between the different presentations and visual outcome at 1 month. Sub analysis of each presentation revealed that patients presenting with diffuse epithelial keratitis were more likely to end up with a vison of 20/40 or better at 1 month. Three patients presented with stromal keratitis (75%) showed reduced visual acuity, which was statistically significant. Two patients that presented with disciform keratitis also showed reduced visual acuity; however, the difference was not statistically significant.

**Table 4:** Visual acuity analysis for different presentations at one month

Presentation	Visual acuity		*Fisher Exact Test* *P-value*	*Chi-square* *P-value*
	20/40 or better	Worse than 20/40		
	%	%		
Diffuse epithelial keratitis	93%	7%	0.02	0.03
Disciform keratitis	0	100%	0.06	
Keratoconjunctivitis	67%	33%	1	
persistent epithelial defect	100%	0	1	
Stromal infiltrate	25%	75%	0.04	

## Discussion

The first published case of ocular microsporidiosis was of an 11-year-old Sri Lankan boy [11]. Historically, microsporidial keratitis was considered a rare ocular complication of HIV/AIDS [5]. In our study, however, all patients were immunocompetent. Increased awareness and advances in diagnostic tools have led to an increasing number of reports of microsporidial keratitis in recent years. In addition to the previously known risk factors, we noticed that 23% of patients used unsterile honey as a topical herbal remedy. Honey can be a major source of microsporidia as it can infect the honeybees [12]. To the best of our knowledge, we are unaware of any previous reports identifying the use of unsterile topical honey as an association with microsporidial keratitis. Another important finding was that over half of patients (54%) used topical steroids in different preparations indicating it as a potential predisposing factor for developing microsporidial infection [13]. However, it remains unclear if the steroids were used pre-infection, as they may have been used to alleviate the symptoms of preexisted microsporidial infection.

The diagnosis was confirmed by identifying microsporidial organisms in corneal or conjunctival scrapings using light microscopy. A key aspect of diagnosing microsporidial keratitis is the morphological identification of organisms in corneal tissue scrapings. The most effective stain is a mixture of 0.1% calcofluor white and 10% potassium hydroxide. The traditional Ziehl–Neelsen staining method with 20% sulfuric acid also stains the organisms effectively. Transmission electron microscopy with a Gram stain reveals intraepithelial, gram-positive, oval-shaped spores [14]. Other confirmatory tests include confocal scanning, optical coherence tomography, impression cytology, and PCR [6].

Patients with microsporidial keratitis often present with foreign body sensation, tearing or dryness, redness, photophobia, and vision impairment. Clinically, almost all studies in the literature have divided microsporidial keratitis into two forms: keratoconjunctivitis and stromal keratitis. The keratoconjunctivitis type usually presents with mild-to-moderate non-purulent conjunctivitis, followed by the development of multiple raised coarse epithelial lesions (stuck on lesions) that take up fluorescein stain. This form only affects the anterior layers of the cornea, except in a few reports where it was associated with uveitis or endotheliitis [15]. This process may take a few weeks to resolve and may subsequently leave a corneal scar or haze. However, all signs may not be present in all patients [16]. In our study, some of the patients with keratoconjunctivitis type presented only with corneal epithelial signs. Owing to its epithelial manifestation, this type may be misdiagnosed as an adenoviral infection related to dryness or contact lens. This type of epithelial keratitis appears to be self-limiting, only requiring symptomatic therapy, according to a prospective randomized clinical trial involving 145 cases and other reports [17]. Despite that, it can have a protracted prognosis with sub-epithelial infiltrates and subsequent scarring [18].

Another clinical pattern of microsporidial infection is stromal keratitis, which can also be difficult to diagnose early as it may resemble herpetic, fungal, bacterial, acanthamoeba infections, or auto-immune keratitis. This is supported by most of our patients being initially misdiagnosed with different types of infections (Table 1). The clinical picture of microsporidial stromal keratitis varied in literature with no clear, consistent, well documented pattern. For instance, one case series of microsporidial stromal keratitis found that the most common corneal picture was multifocal stromal infiltrate with surrounding edema [9]. This picture could easily be misinterpreted as bacterial or fungal keratitis. Another study showed that 67% of stromal keratitis cases presented with a disciform keratitis picture [19].

In addition, three patients presented with a picture of a persistent epithelial defect. We are unaware of any previous reports linking this unique presentation of microsporidial infection to microsporidiosis. Unlike herpetic keratitis, where the sensation is typically affected, the sensation remained intact in these cases. Careful interpretation of these presentations suggests that microsporidia can affect corneal layers in a pattern very similar to herpetic keratitis. Microsporidia can affect the epithelium, stromal, and endothelium. Moreover, stromal keratitis due to microsporidia can present with presumably immune related interstitial keratitis, similar to interstitial keratitis secondary to herpes [20]. The published data also revealed limited cases of uveitis and endophthalmitis caused by microsporidia. Thus, the classification of microsporidia infection should be adjusted based on the specific layer of cornea affected and whether it is an immune or infective type. This novel classification (Table 2) will allow for greater understanding, earlier recognition, and better assessment of this infection. However, further research is needed to support this classification.

The efficacy of treatment regimens in eliminating the infection was demonstrated by our data. Each patient received both fumagillin and fluconazole eye drops for 1 month. In the literature, medications such as albendazole, thiabendazole, itraconazole, fumagillin, propamidine isethionate, chlorhexidine, metronidazole, polyhexamethylene biguanide, benzimidazoles, and fluconazole have been used to treat microsporidial keratitis with variable outcome [14]. In our series, visual acuity improved at 1-, 3-, and 6-months of follow-up. However, cases with stromal infiltrates and disciform keratitis like picture showed inferior visual outcomes, and one fourth of stromal keratitis cases required surgical intervention such as keratoplasty. All patients remained infection-free post-treatment with no episode of recurrence at 1 and 6 months. Only one case resulted in significant corneal scarring. The limitations of our study includes the retrospective nature, small sample size, missing follow-up especially for 6 month visits, and the lack of a comparative group. In conclusion, microsporidial keratitis has different presentations and can be easily misdiagnosed initially which may result in delay in diagnosis and poor outcome. Risk factors included contact lens wear, topical steroid use, and unsterilized honey use. Newer classification is suggested to facilitate early diagnosis and prompt treatment. Most of the cases resulted in favorable outcome on medical treatment except the stromal keratitis cases.

## Data Availability

No datasets were generated or analysed during the current study.
